# Traits of Complex Thinking: A Bibliometric Review of a Disruptive Construct in Education

**DOI:** 10.3390/jintelligence10030037

**Published:** 2022-06-30

**Authors:** Jose Jaime Baena-Rojas, María Soledad Ramírez-Montoya, Diego Mauricio Mazo-Cuervo, Edgar Omar López-Caudana

**Affiliations:** 1 Institute for the Future of Education, Tecnologico de Monterrey, Monterrey 64849, Mexico; jose.baena@tec.mx (J.J.B.-R.); solramirez@tec.mx (M.S.R.-M.); 2 Fundación Universitaria CEIPA, Sabaneta 055450, Colombia; diego.mazo@ceipa.edu.co

**Keywords:** complex thinking, epistemology, bibliometrics, educational innovation, higher education

## Abstract

The purpose of this research is to contextualize the behavior of publications on complex thinking in education. A total of 428 documents compiled in Scopus from 1937 to 2022 were analyzed with a bibliometric study considering criteria such as “complex thinking”, “complex thought”, and “reasoning for complexity”, all combined with education. The results show 153, 47, and 5 publications for each criterion with their related disciplines, citations, types of documents, universities, prominent authors, researching countries, and the general diachronic evolution of the subject, this allows to establish an idea about the implications of the present study according to one of the most important databases in the world. It is concluded that complex thinking and its relationship with education awakens a greater interest in the academy, not only because of its incidence in diverse fields that are nourished by it for the generation of new multidisciplinary knowledge but also because of the published research that demonstrates its transcendence.

## 1. Introduction

Complex thinking as a construct within epistemology alludes to the nature of associativity between a series of elements that unfailingly share an essential complementarity. Thus, although such elements may be heterogeneous, in any case, there is a dynamic relationship between all of them, which represents a paradox of the one and the multiple in systemic terms. Therefore, complexity is, by default, the filigree of events, interactions, retroactions, and other phenomenal circumstances that grow and integrate the world (Morin 2005; Moraes and Petraglia 2021).

Thus, complex thinking brings together, from a systemic approach, the different interactions between objects, people, and the environment at a holistic level, which successively impacts knowledge. The latter must be considered in an integral and not fragmented way, all in order to achieve awareness that even the social system is in constant interaction with the diachronic reality and therefore depends not only on technology but also on the progress that humanity itself tends to achieve over time (Pereira 2010).

In this sense, complex thinking concerns not only part of the process of thinking and understanding reality but also the way in which people manage uncertainty from learning and education. The above, because it is from education that people have partially been able to solve adversities as well as different problems of life, trying to give concrete answers to the infinite questions that from the phenomenal surround him, questions that are also presented as binding elements to continue understanding life and the environment (González Velasco 2018).

Even from education itself, in whatever discipline it may be, it becomes necessary to establish other ways of understanding the construction of subjects in the world, and the ideal is starting from the incorporation of the dimensions of knowledge and directing them from the totality of what the human being can learn. In this sense, education becomes determinant and must, epistemologically speaking, have precise tools that show people new paths for the development of skills from the complexity that surrounds them; thus, allowing a better interpretation of reality (Santos 2000). Learning experiences, creative autonomy, and the current context will be determining elements for future systemic transformations. Because of this, the notion of complexity makes possible the debate within the educational tradition and the elements of change to practical reflexivity around educational processes in general that involve new cognitive horizons regarding the teaching of different disciplines (Ferrada Sullivan 2018).

In this way, complex thought tends to take a notable and significant relevance within the becoming of epistemology as well as within multiple scientific fields and disciplines, including educational sciences. Therefore, the main objective of this article is to contextualize the behavior of publications on complex thought and other known variants, such as complex thinking and reasoning for complexity. All of this is considered in the frame of education through a bibliometric analysis.

It is important to note that bibliometric reviews matter in research because all of these can show the interests of some universities which currently work on some specific fields to delve into some trending topics or even produce new knowledge. In this way, a universities’ publications represent one of its greatest intangible assets because all of them show the impact of these higher education institutions on society at a national as well as an international level for solving problems or explaining. Likewise, these publications can be construed as an effective, relevant, and above all, a disruptive means for emerging into academia as a potential leader in some scientific disciplines where universities and some research centers are investing resources and outstanding efforts. Therefore, bibliometrics describe publication patterns, using quantitative analysis and statistics, which in some cases inspire other universities and institutions for working and moving forward with new articles, books, conference proceedings, among other publications, influenced by subsequent research by others. Bibliometrics can be decisive for identifying how many articles universities and research centers have published or even how many all of them have assessed, then using citation analysis to look at how those scientific papers are impacting new research. Counting publications can be useful for doing some comparisons, but a citation analysis grants researchers and academia a look at the impact those articles have had on others by determining how often they are considered. A citation analysis can also reveal what journals, universities, scientific organizations, and even countries have a remarkable impact in different fields of research (McBurney and Novak 2002; Wallin 2005; Belter 2014; Rüdiger et al. 2021).

Therefore, as the main contribution, the current study reveals the leaders’ regions, universities, and even authors who currently work in the indicated field to produce new knowledge in this regard. Mainly, this is because some specific topics can become intangible assets, increasing the recognition of universities and research centers when all of them produce new scientific research for other researchers interested in better understanding and explaining some phenomena depending on the study which is being dealt with. Likewise, the paper will delve into aspects such as the origin of the concept, the total number of publications, related disciplines, citations, types of documents, universities, prominent authors, and the regions where most work is conducted and published on the subject under study.

## 2. Literature Review

### 2.1. Origins and Reflections on Complex Thinking

Knowledge is completely dynamic and adapts itself to the circumstances in time as well as revolutions that are reached at certain moments. For this reason, the confrontation of the sciences with the truth is a praiseworthy attempt to achieve the best possible interpretation of reality (Tsoukas 2005). All this is obviously according to the available instruments provided by human ingenuity to achieve the immanent purpose of understanding it. Hence, knowledge is only one element of a more general system, where education and didactic strategies to facilitate the solution of problems can be the effective means to achieve the assimilation of all the complexity involved in explaining the world from the sciences and humanities (Ryoo and Linn 2015; Sigahi and Sznelwar 2022).

Thus, and considering the different revolutions of knowledge and, with this, the various changes in the Western paradigm that involved a remarkable reductionism in the separation of knowledge during the twentieth century, the French philosopher Edgar Morin ends up precisely proposing the construct of complex thinking, whose purpose is to articulate the scientific culture, the culture of the humanities, and the artistic culture in a transdisciplinary way. This is based on the integration of ideas, concepts, and notions derived from different theoretical sources. What has led to complex thinking is to experience a dialectical confrontation that has been leaving an original legacy of social and rational transformation within the theory of knowledge (Marshall 2016; Barberousse 2008).

Obviously, complex thinking focuses on formulating questions that attempt to address the reality of phenomena, whatever it may be, and that has to do with the way in which people interact with it, rather than arriving at the answer because complexity involves an imperfection that will always unfailingly be accompanied by uncertainty as well as the recognition of the irreducible. Likewise, although simplification is essential in most cases for general understanding, it must be accompanied by relativization because it can be deliberately accepted in order to better assimilate any event or phenomenon but not to reduce it because it is believed that the absolute truth has been reached. In other words, complexity is the union and interaction of the simple and the complex, where hierarchization, disintegration, and the reduction in different aspects integrate a whole that, at the same time, possesses stages from the infinite mental compression (Morin 2005).

The very notion of what is understood by complex thinking is as dynamic as the raison d’être of what it tries to explain; in fact, it can be said that there is no exclusive and unique definition for which it is also considered a construct. It is for this reason that both the knowledge revolution and the scientific–technical revolution continue to advance, and with them, new questions are raised, as is necessary in this case with the social sciences (see Table 1), about the role of the different forms of knowledge and the way in which they interpret the world. In other words, complex or complexity thinking is concerned with reconciling the classical perspectives of thought, which persist, with the new quantum-relativist and scientific–technical revolutions (Delgado 2002).

Thus, considering all the previous ideas, it can also be added that one of the most significant moments for the human being, at a phenomenal level, is their own daily life, where an infinity of facts, circumstances, relationships, and experiences converge, fluctuating between the micro and macro world, which one seeks to interpret in order to build a worldview of reality. In this way, daily society life through education is not only transmitted from generation to generation but evolves together with the new tools offered by technology, such as the internet, information communications technologies (ICT), software, artificial intelligence (AI), robots, devices, among a long list that grows every single year. All of them positively impact the performance of complex thinking, in general, as well as education from a troubleshooting perspective (González Velasco 2018; Marshall 2016; Baena et al. 2017; Yanchar et al. 2017).

### 2.2. Complex Thinking and Education

Clearly, complex thinking is a determinant for the sciences in general because it induces those who research and teach to seek new learning skills that adapt both to the realities of knowledge in permanent construction and to the realities that are also present within the educational process. In other words, the daily work of learning and teaching should not decline in the face of the need to rethink scientific knowledge and the imperative of making use of new techniques to solve problems that subsequently also involve the implementation of new values and learning according to the circumstances (Álvarez Nieto 2016).

**Figure 1:**
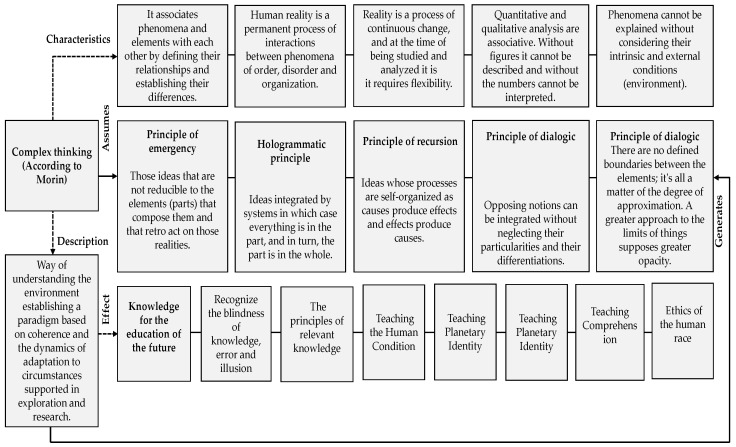
Some characteristics of complex thinking. Source: own elaboration based on Salcedo (2014).

There is also an imperative to readapt the way in which people process factual and non-factual facts, so that this processing coincides with the characteristics of complex thinking, see Figure 1, and therefore the same is more in line with the needs of society, including in education. This situation requires the integration of all knowledge, considering the separation that was established during the twentieth century within the different scientific specialties as well as within the academy itself. Therefore, all this integration previously mentioned constitutes a complementation process that may end up resulting in the cognitive and interpretative improvement of reality (Varona Domínguez 2020).

Therefore, it can be said that complex thinking is the result of the new scientific paradigm, which was built in the mid-twentieth century and whose initial theoretical contributions were intended to clarify systemic behaviors that were anomalous in principle. In any case, although complex thinking is more typical of the exact sciences, all its methodological postulates have also been implemented within social systems, as well as within the different forms of social organization (Vásquez and Viguri 2020).

Nowadays, philosophers and experts in different fields of science are increasingly reflecting on the modes of perception that generate historical knowledge and its adaptations to the reality of the present itself within the culture of the different social organizations. This implies the need to include the notion of complex thinking as a new type of rationality considering its trans-disciplinarity and influence in the most holistic context for decision making that generates precedents in a kind of co-evolution, both for learning and for education itself (Chernikova 2015; Chekantseva 2017).

In fact, the trend is that more and more new studies on complex thinking applied in the field of education are being developed (not only because of the interest of the academy itself but also because of the disruptive implementation of technological tools). These not only seek to analyze, from the logic of cognitive processes, the way in which students understand the daily interactions with other people and the world, but also how they, from their abilities, solve certain problems that may arise at the level of social interaction. Therefore, it can be said that studies on complex thinking consider the learning and reasoning achieved on academia that clearly lies in the competencies, values, and tools that education itself offers to face various challenges and problems (Ovens et al. 2013; Smith and Knowles 2017).

It is for all these reasons that complex thinking is dynamic and clearly, from education, plays a determining role in overcoming important challenges related to the strengthening of reasoning competencies for complexity based on critical thinking and creative thinking. Therefore, it is undeniable that literature reviews and bibliometric studies can also influence the evaluation and interpretation of specific thematic areas within scientific research (Ramírez-Montoya et al. 2022).

## 3. Materials and Methods

In this section, the authors define the adopted techniques for processing and, above all, for analyzing the information and databases consulted according to main purpose of the entire paper. Hence, the research question of this study aimed to answer is: What is the behavior of publications in complex thinking within education considering its influence as a potential intangible asset for universities and research centers?

Then, this current article carries out a bibliometric review where documents such as scientific articles, books, book chapters, and conference proceedings, among other publications, are identified through the Scopus database. The above allows to know the development of new contributions regarding complex thinking in education, in addition to its evolution over time concerning certain topics and publications. This denotes the interest of the academy to work more in depth with a general and comprehensive view of any analyzed phenomenon, just like that which happens in this study with the behavior of publications in complex thought (Donthu et al. 2021).

Thus, Elsevier’s Scopus was consulted; it is considered one of the largest and most prestigious databases of citations and peer-reviewed literature, launched to the public in 2004 and whose content provides a comprehensive summary of research results worldwide in various fields of science, including technology, medicine, social sciences, and humanities (Ball 2021).

Similarly, the present research completes a bibliometric process defined as a segment of scientometrics that applies mathematical and statistical methods to any type of scientific literature, as well as to the authors that generate it, to study and analyze the behavior around the production of new knowledge. This is also understood as a study of productivity, which involves creative methodologies and a conceptual structure for the creation of research metrics, which are subject to both the financing, transformation, generation, and impact of such publications that ultimately represent new trends as well as scientific contributions regarding what is being researched nowadays (Cortés Vargas 2007; Marais and Meylaerts 2022). On the other hand, the research also completes a descriptive process, which alludes to the analysis that characterizes any phenomenon or occurrence in various disciplinary fields as in this case with the field of social and human sciences. Thus, this methodology allows to deduce a situation that is presented (in this case from bibliometrics); considering also all its dimensions, due to the present proposal focuses on collecting data that describe the situation as it essentially manifests itself (Lahitte et al. 2010; Shields 2020).

**Figure 2:**
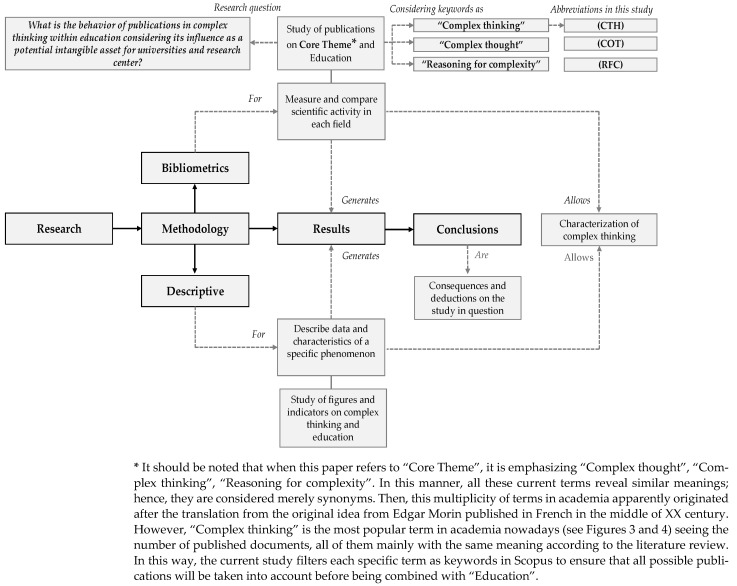
Processing of information from the methodological approach. Source: own elaboration based on Cortés Vargas (2007) and Lahitte et al. (2010).

The search for information in Scopus was completed using technical terms such as “complex thinking (CTH)”, “complex thought (COT)”, and “reasoning for complexity (RFC)”, all of which are also combined with “education (EDU)”. These concepts were also searched in the English language in order to guarantee the coverage of documents in the database under study. After verifying the content of the documents with the indicated terms, we proceeded to process all this information with the indicated methodology as shown in Figure 2.

Similarly, the total number of publications, related disciplines, and citations were then determined as the behavior over time of this notion as other bibliometric works usually characterize publications in a given field (Baena-Rojas and Alzate-Rendón 2020). Then, the origin of such publications as the regions where most work is currently being conducted and, subsequently, the types of documents, citations, universities, and most prominent authors from the published literature that specialized in the topic of complex thinking and education were determined.

## 4. Results

**Figure 3:**
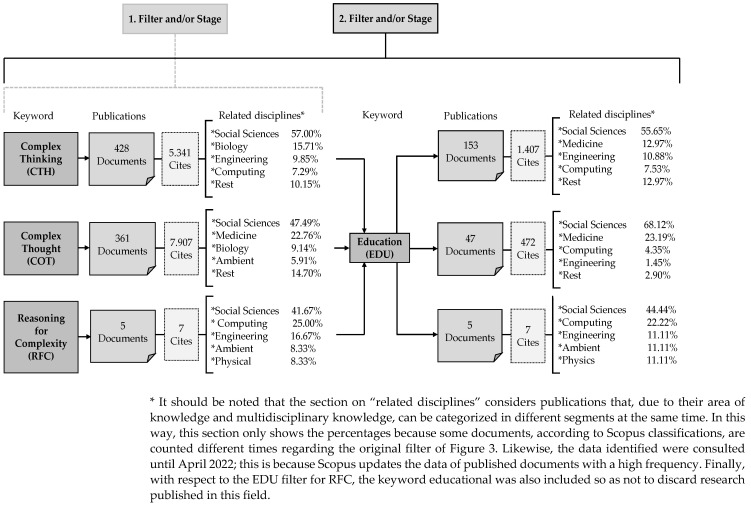
Documents and citations within the field of complex thinking and education. Source: own elaboration based on Scopus (2022).

In the first part of this section, the total number of documents tracked in Scopus according to the keywords previously defined and recognized are characterized, see Figure 3. For the case of “complex thinking (CTH)”, a total of 428 documents with 5341 citations; for the case of “complex thought (COT)”, 361 documents with 7097 citations; and for the term “reasoning for complexity (RFC)”, 5 documents with 7 citations. The above, then, implies that these are usually the most recurrent terms to refer to the subject of study of this work and that there is also a relationship between this multidisciplinary construct with other fields, mainly with the social sciences.

Figure 3 also indicates that when the additional term “Education (EDU)” is included in the filter for each of the previous potential synonyms of complex thinking, a smaller number of 153 documents and 1407 citations are generated for the first term, 47 documents and 472 citations for the second term, and then, after the filter, the 5 documents and 7 citations are maintained for the third term which seems to be directly linked to the field of education. After this new filter, it is evident, despite the decrease in research, that complex thinking in general within education maintains a more notable relationship with the social sciences concerning other disciplines.

**Figure 4:**
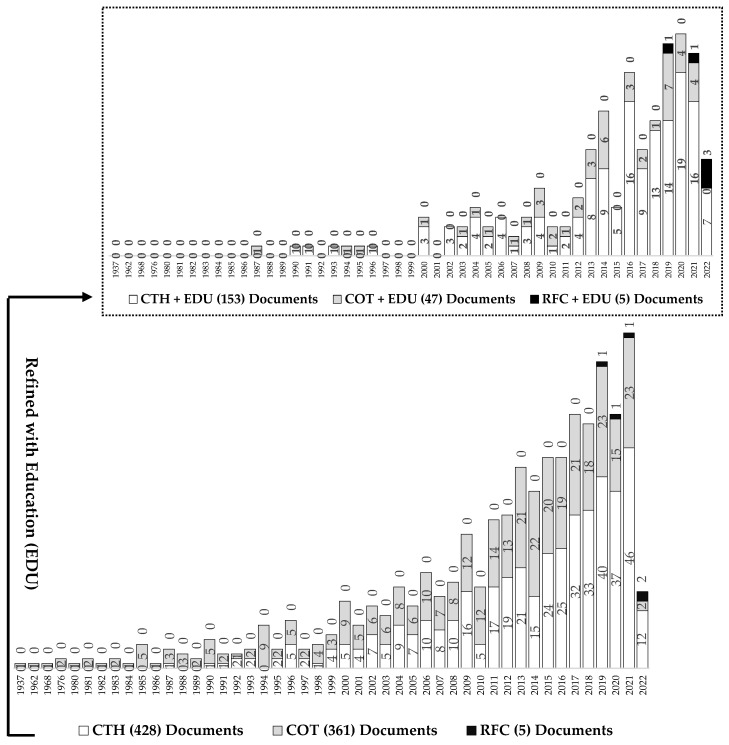
Historical behavior and variation of publications on complex thinking and education. Source: own elaboration based on Scopus (2022).

The second part shows the behavior over time of this notion, see Figure 4, where the investigation on central theme of the study in Scopus began in the first half of the 20th century, in 1937, specifically with the term “complex thought (COT)” and with higher notoriety compared to “complex thinking (CTH)”; the latter had publications only until 1987. Nevertheless, to date, there has been a considerable increase in the publications of “complex thinking (CTH)” that began in the 21st century during the year 2000 and finally exceeded the total of the works on “complex thought (COT)” despite this last term starting before in the publication of scientific documents. Similarly, and specifically for the field of “reasoning for complexity (RPC)” within the central theme of the study, publications barely begun in 2019, which suggests then that this research topic is new, and new contributions are just beginning in this regard.

Similarly, Figure 4 shows that the number of papers on the areas of “complex thinking (CTH)” and “complex thought (COT)” when combined with the area of “education (EDU)” tends to decrease to less than half of the original publications. However, when combining the “reasoning for complexity (RFC)” domain with “education (EDU)”, the number of publications remains the same as the original, decreasing by only one publication, which suggests that this domain has a close relationship with education, which is why there are no changes when refining.

**Figure 5:**
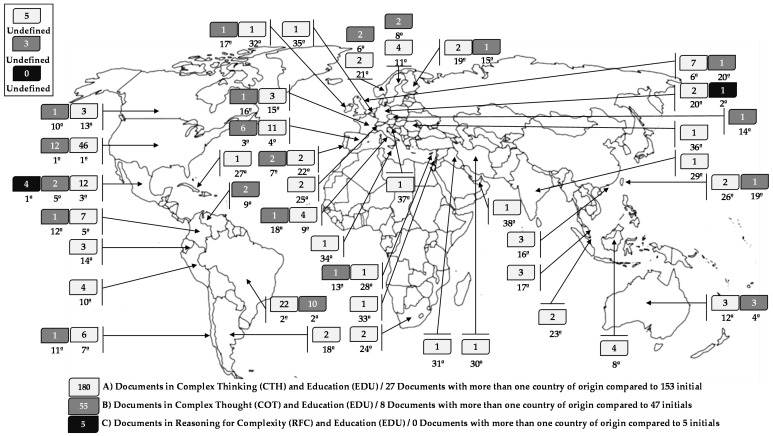
Origin of documents on complex thinking and education. Source: own elaboration based on Scopus (2022).

In the third part, the origin of such publications is determined by country and by the regions where most work is conducted on the central theme of this work, focusing on the areas of “complex thinking (CTH)”, “complex thought (COT)”, and “reasoning for complexity (RFC)”, each of these combined with “education (EDU)”. It is important to note that the number of papers, see Figure 5, does not coincide with most of the total publications previously indicated, (153 and 47 papers—except the last case of 5 papers) because Scopus counts the origin of the papers according to the origin and the number of authors of the papers, who evidently can be from different places. This means that the same document can be registered several times in the database to recognize the origin of one or more authors who may originate from different countries.

Thus, according to Figure 5, the countries with the largest number of publications in the areas of “complex thinking (CTH)” and “education (EDU)” are the United States with 46, Brazil with 22, and Mexico with 12 publications, equivalent to 25.56%, 12.22%, and 6.66%, respectively, of this new total, which includes documents with more than one country of origin. In the area of “complex thought (COT)” and “education (EDU)” are the United States with 12, Brazil with 10, and Spain with 6 publications, equivalent in this case to 21.82%, 18.18%, and 10.91%, respectively. Finally, for the area of “reasoning for complexity (RFC)” and “education (EDU)”, the country with the most publications is Mexico with four followed by Germany with one, equivalent to 80% and 20%, respectively.

In the fourth part of this section, for each combination of fields, the types of documents, the total number of citations, the universities that developed the documents (articles, conference proceedings, books, etc.), and the most prominent authors, within the framework of the central theme of this research, are recognized. Thus, the results show that, see Table 2, in the case of “complex thinking (CTH)” and “education (EDU)”, the documents are specifically articles with 104, conference proceedings with 31, book chapters 8, books 2, and other 8 works, equivalent to, respectively, 67.97%, 20.26%, 5.23%, 1.31%, and 5.23%. In the case of “complex thought (COT)” and “education (EDU)”, the papers are articles with 39, conference proceedings with 3, book chapters with 1, books 2, and others with 2 papers, which are equivalent to, respectively, 82.98%, 6.38%, 2.13%, 4.26%, and 4.26% papers. Then, for the case of “reasoning for complexity (RFC)” and “education (EDU)”, papers are articles with two, conference proceedings with two, and others with two, which subsequently equal 40%, 40%, and 20%.

Table 2 also shows how, with the filter “complex thinking (CTH)” and “education (EDU)”, there are 1407 citations in Scopus. Then, when analyzing the most cited documents, it is identified that they come mainly from the United States universities, such as Stanford University, University of California, University of Connecticut, Claremont McKenna College, and University of Maryland, among other universities that join the top five. With the filter “complex thought (COT)” and “education (EDU)”, there are 472 citations which, when analyzing the most cited document in this category, comes from Vanderbilt University in the United States with a total of 184 citations, in addition to the other universities that complete the ranking of the top five. Finally, with the filter “reasoning for complexity (RFC)” and “education (EDU)”, there are only seven citations, which come from Friedrich–Alexander University Erlangen and the International School of Management, both from Germany, as well as from Tecnológico de Monterrey in Mexico, which shows how recent this last field is within the central theme of this article.

**Figure 6:**
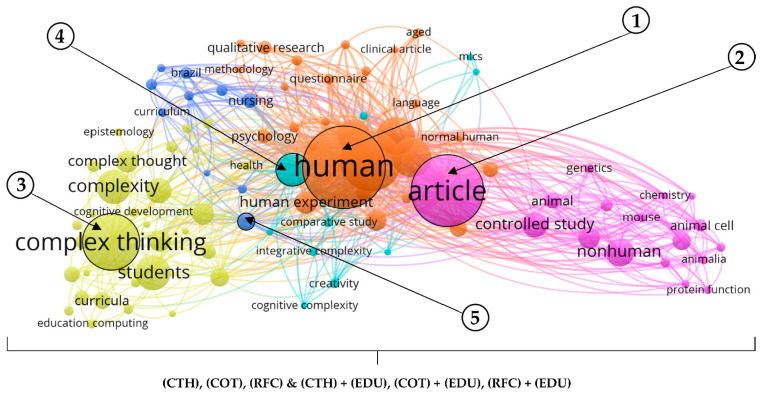
Co-occurrence analysis for all papers selected for this study from 1937 to 2022. Source: own elaboration based on Scopus (2022).

In the fifth and last part of this section, it is evident that there are some interesting patterns among all the papers and published research on Scopus. In this case, the graphical representation of the co-occurrence networks shows the inferences and relationships between entities, terms, and in general, the most relevant words according to their frequency in the works obviously considered in this study. In other words, according to previous identified criteria, see Figure 3 and Figure 6 reveals the most recurrent matters within the documents considered for this bibliometric study.

Then, considering a minimum number of seven occurrences per 100 keywords, it is possible to point out that there are five clusters related with the terms most repeated from the set of data downloaded from Scopus. The first, in this case, “human”, with 951 total links strength, 152 occurrences, and 88 links. The second, “article”, with 770 total links strength, 126 occurrences, and 83; the third, “complex thinking”, with 179 total links strength, 93 occurrences, and 49 links. The fourth, “thinking”, with 319 total links strength, 46 occurrences, and 65 links, and the fifth, “learning”, with 132 total links strength, 22 occurrences, and 52 links. Thereby, the evidence shows that recurrent patterns exist in the analyzed papers, for instance, the most relevant matters by co-occurrence and their relationships with others. All of this, when academia considers “complex thinking (CTH)”, “complex thought (COT)”, “reasoning for complexity (RFC)”, and “education” (EDU) as the designated criteria in this case.

In this manner, Figure 6 also indicates that, in the first case, within “human”, some relevant subthemes exist, such as humans, male, and female with 693, 501, and 408 total links strength, respectively. In the second case, with “article”, there are relevant subthemes such as controlled study, nonhuman, and animals with 259, 234, and 195 total links strength, respectively. In the third case, within “complex thinking”, there are relevant subthemes such as students, decision making, and education with 178, 152, and 144 total links strength, respectively. Subsequently, in the fourth case, with “thinking”, there are subthemes such as psychological aspect, comparative study, and intelligence with 73, 68, and 38 total links strength, respectively. Lastly, in the fifth case, “learning” highlights some subthemes such as clinical competence, nursing education, and medical education with 78, 78, and 70 total links strength, respectively.

## 5. Discussion and Conclusions

Bibliometric studies are gaining more and more importance due to the development of new computer tools and scientific databases such as Scopus. Thus, epistemologically speaking, complex thinking as a construct is evidence of a constant process of development since its origins, starting in the mid-twentieth century. This is not only because its multidisciplinary nature enriches its raison d’être but also because it is usually interpreted as a paradigm of knowledge and/or a type of rationality that can favor the way phenomena or occurrences are approached in general to understand them and provide answers to the problems they entail according to the historical context. All of the above becomes congruent with what some theorists argue regarding why complex thinking and education present more and more research over time, a situation that supposes a progressive interest within the academy to understand the subject in more depth (Ovens et al. 2013; Smith and Knowles 2017; Donthu et al. 2021), as seen in Figure 4, Figure 5 and Figure 6 and Table 2.

While it is true that complex thinking brings together various fields of scientific knowledge precisely because of its conditional and multidisciplinary nature, the present work reveals that, according to the scientific literature itself, most of the publications on this topic come mainly from the field of social sciences with other thematic connections as reflected in Figure 3. Therefore, it can be stated that this relationship of complex thinking with the field of social sciences stems from the possibilities that the latter offers to complement the different branches that integrate it and whose purpose is to analyze human behavior and the different interactions with its reality and environment.

Education as a process to promote learning and strengthen the mastery of certain knowledge, skills, values, beliefs, and other key elements to understand reality is an essential aspect to deepen and expose the role that complex thinking can have in the acquisition of skills and competencies within the sciences in general. Therefore, there is a huge potential for growth in the creation of new research and literature that specializes in the use of complex thinking to improve didactic and learning processes in education in general, considering the interest of the academy to enhance in this section of educational innovation, considering the constant advances in information and communication technologies, as well as the possibilities offered by the latest industrial revolutions.

All of the above is merely related to the position of McBurney and Novak (2002) who state that scientific organization and university publications are one of its greatest intangible assets. Obviously, this is because this new and corroborated knowledge can catapult these institutions to the cutting edge of scientific and technological knowledge around the world as well as improving their metrics for international rankings. Hence, these are some critical reasons why bibliometrics matter in order to measure the behavior of publishing scientific papers and their impact within academia.

Finally, at present, according to the search criteria in Scopus, the main countries that lead the production of complex thinking and education are the United States, Brazil, and Mexico, concentrating together almost half of the world’s production with 44.44% in this subject, where this production also corresponds to 67.97% of articles, 20.26% of conference proceedings, 5.23% of book chapters, and the remaining 6.54% of other types of documents. Likewise, in the case of complex thought and education, the countries that stand out are the United States, Brazil, and Spain with 50.91% of the publications, where 82.98% of the production corresponds to articles, 6.38% to conference proceedings, 2.13% to book chapters, and 8.51% to other types of documents.

It is striking that the main countries that lead the production of scientific papers in the main object of this study are currently in the top of the research and development expenditure (% of GDP) of their regions. This proves, according to Baena Rojas et al. (2020), that a clear incidence exists between the state budget and scientific research production based on a potential budget for public policies in this regard. Although, in Mexico’s case, this research and development expenditure (% of GDP) is less than one percent, and lower regarding some countries of its region; this does not mean that the same pattern is similar for private universities because some of them are leading in the production of scientific papers in complex thinking. In fact, regarding this last analyzed country, in the case of reasoning for complexity and education, the countries that stand out are Mexico and Germany as the only producers of literature on this subject, with 80% and 20%, respectively, where this production corresponds to 40% of articles, 40% of conference proceedings, and the remaining 20% of other types of documents.

## Figures and Tables

**Table 1:** Notions of complex thinking in some social science approaches *.

Approach	Notions	Source
Anthropology	In this social science, there have also been relevant changes in the logic of complex thinking. This is because the method of simplifying has been rethought because the whole is now not the sum of the parts but the relationship between all of them. It is for all this that the object of study of human reality and even the ethnographic techniques of this discipline try to relate the different levels of reality and knowledge, to reach a complex and global vision of society and its cultures.	(Fortunato and Porto 2021; Solana Ruiz 2011)
Political Science	This social science based on a traditional paradigm of simplicity tends more and more to adapt to social reality. In this case, technological advances, the impact of the mass media, the relationship between the State and society, as well as other factors external to the discipline, show the complexity of politics and human organizations. Thus, the limitations of the scientific method imply the need to establish new paradigms and a revolution of knowledge within this discipline. Consequently, contemporary political science must be approached from a multidimensional or complex focus, which allows for a broad approach to the political phenomena of the present, because they are developing rapidly, nurturing the theoretical and methodological relativism demanded by this field.	(Gallego and Arteaga 2013; Osorio García 2012)
Law	As a discipline this includes not only the values, principles, and moral convictions contained in constitutions, laws, jurisprudence, and other legal systems adaptive to certain contexts. In this sense, law manifests an open and flexible essence precisely because, from the complex thought, it intends to link and transmit the subject’s commitments and social conscience from their cultural imaginary, language, and norms. Therefore, from complexity, it supposes an alternative perspective as holistic of the nature of modern society characterized by being an intensive combination of law, politics, economics, and other social disciplines.	(Pinilla-Rodriguez et al. 2017; Morin 2005)
Economy	This discipline is possibly one of those that has been adapting the most to the current of complex thinking, given that from its perspective, it modifies, intervenes, and transforms the administration of scarce resources, characterized by the complexity of its phenomena. Thus, the economy achieves theoretical models that use several variables and logical relationships between them, which represent the functioning of economic processes. The above is, without ignoring the holistic, where uncertainty is constant in human behavior. It can be said then that there is a relationship between progress and scientific and technological advances, which results in prosperity, wealth, and economic growth within societies.	(Caro Ramírez 2021; Guzmán Díaz and Adriano Anaya 2013)
Sociology	At present, certain authors of contemporary sociological theories consider globalization as the cornerstone of their analysis, questioning the heuristic potential of the approach in classical sociology, thus managing to place complex thinking at the center of their conceptual approaches. In other words, this concept and other related concepts, such as uncertainty and irreversibility, delimit the new paradigm shifts within scientific knowledge which reached an evolution and development at the epistemological level of this nature in the field of classical sociological theory.	(Bonacci 2013; Wallerstein 1999)

* Although all fields of knowledge are analyzed in this research. In this case, a specific example is applied to this branch of knowledge focused on the study of society and human behavior. Source: own elaboration based on Zoya and Aguirre (2011).

**Table 2:** Types of publications and other relevant features on complex thinking.

Areas	Type of Documents	N.	Cites	Main Universities		Authors	Cites	Sources
CTH + EDU	Article	104	1.407	1º	Stanford University/University of California/University of Connecticut/Claremont McKenna College/University of Maryland	(Antonio et al. 2004)	319	Psychological Science
	Conference Proceedings	31		2º	Bowling Green High School	(Muir-Herzig 2004)	99	Computers and Education
	Book chapter	8		3º	Temple University/Pennsylvania State University	(Shapiro and Stefkovich 2016)	79	Ethical Leadership and Decision Making in Education: Applying Theoretical Perspectives to Complex Dilemmas: Fourth Edition
	Book	2		4º	University of Missouri	(Greening and Johnson 1996)	65	*Journal of Management Studies*
	Others	8		5º	University of Illinois at Chicago	(Morales-Doyle 2017)	59	Science Education
	**Total**	**153**			Rest of universities		786	Others
COT + EDU	Article	39	472	1º	Vanderbilt University	(Desimone and Le Floch 2004)	184	Educational Evaluation and Policy Analysis
	Conference Proceedings	3		2º	The Australian National University Australia	(Byrne et al. 1995)	123	*Journal of Psychosomatic Research*
	Book chapter	1		3º	Undefined	(Hillel and Miller 1987)	16	Advances in experimental medicine and biology
	Book	2		4º	University College Dublin/University of Trás-Montes e Alto Douro	(Ahern et al. 2019)	15	*Studies in Higher Education*
	Others	2		5º	University of Alberta	(Brindley and Dunn 2009)	11	*Journal of Critical Care*
	**Total**	**47**			Rest of universities		123	Others
RFC + EDU	Article	2	1	1º	Friedrich-Alexander University Erlangen/International School of Management	(Schott et al. 2020)	6	*Journal of Manufacturing Technology Management*
	Conference Proceedings	2		2º	Tecnológico de Monterrey	(Ramírez-Montoya et al. 2021)	1	ACM International Conference Proceeding Series
	Book chapter	0		3º	Tecnológico de Monterrey	(Ramírez-Montoya et al. 2022)	0	*Journal of Open Innovation: Technology, Market, and Complexity*
	Book	0		4º	Universidad Autónoma de Nuevo León/Tecnológico de Monterrey	(González-Pérez and Ramírez-Montoya 2022)	0	*Sustainability (Switzerland)*
	Others	1		5º	Tecnológico de Monterrey	(Rodriguez-Gallegos 2019)	0	Proceedings—IEEE 10th International Conference on Technology for Education
	**Total**	**5**			Rest of universities		7	Others

Source: own elaboration based on Scopus (2022).

## Data Availability

Not applicable.
